# COVID-19 Risk Perception, Trust in Institutions and Negative Affect Drive Positive COVID-19 Vaccine Intentions

**DOI:** 10.3389/ijph.2022.1604231

**Published:** 2022-04-11

**Authors:** Sanjin Musa, Seila Cilovic-Lagarija, Ariana Kavazovic, Nina Bosankic-Cmajcanin, Alberto Stefanelli, Nadia Aleyna Scott, Martha Scherzer, Zsolt Kiss, Katrine Bach Habersaat

**Affiliations:** ^1^ Institute for Public Health of the Federation of Bosnia and Herzegovina, Sarajevo, Bosnia and Herzegovina; ^2^ Sarajevo School of Science and Technology, Sarajevo, Bosnia and Herzegovina; ^3^ Department of Psychology, International University of Sarajevo, Sarajevo, Bosnia and Herzegovina; ^4^ ZK Analytics, Oxford, United Kingdom; ^5^ Centre for Sociological Research, KU Leuven, Leuven, Belgium; ^6^ WHO Regional Office for Europe, Copenhagen, Denmark

**Keywords:** risk perception, vaccine hesitancy, COVID-19, vaccine, trust, affect, behavioural insights, vaccine intentions

## Abstract

**Objectives:** To investigate country-specific drivers and barriers of positive COVID-19 vaccine intentions in the Federation of Bosnia and Herzegovina (FBiH), one of the two entities comprising Bosnia and Herzegovina.

**Methods:** A cross-sectional study design was used, using an online behavioural insights survey tool adapted to the context of FBiH. Three survey waves, each including approximately 1,000 adults, were conducted in July, September and December 2020. Fixed-effects regression analysis was used to explore the drivers, barriers and attitudes towards accepting a future COVID-19 vaccine.

**Results:** COVID-19 risk perception, trust in health institutions and negative affect were positive predictors of positive COVID-19 vaccine intentions, as were living in urban areas and having a college education (versus having primary or secondary education). Conversely, being female, feeling that the pandemic was overhyped by the media and the country of vaccine production were negative predictors.

**Conclusion:** This study provided snapshots on the state of attitudes regarding a future COVID-19 vaccine acceptance and hesitancy in 2020. These findings provided useful insights into the efforts to introduce and roll out the COVID-19 vaccines in FBiH. Further efforts should focus on better understanding the demographic, cultural and behavioural contexts of COVID-related vaccination perceptions in FBiH.

## Introduction

In December 2019, a novel coronavirus disease, COVID-19, emerged [1] and subsequently spread globally. As of 30 April 2021, almost 200,000 cases have been verified in Bosnia and Herzegovina [2], which has a population of approximately 3.3 million. Vaccination remains a critical means in efforts to prevent transmission of the virus, but requires substantial uptake by the population. As many countries globally—including Bosnia and Herzegovina—reported challenges related to population vaccine hesitancy prior to the current pandemic [3], this indicates that successful containment of COVID-19 is likely to involve understanding and addressing vaccine concerns and hesitancy.

This has implications for the introduction of the COVID-19 vaccine in the Federation of Bosnia and Herzegovina (FBiH), one of two entities comprising Bosnia and Herzegovina, where routine vaccination coverage has been decreasing over recent years and is consistently below regional benchmarks [4]. In the period from 2014 to 2018, there was a marked decline in vaccine uptake for numerous vaccines, notably for the measles-mumps-rubella (MMR) vaccine (from 89.1% to 68.4%) and for the diphtheria-tetanus-pertussis (DTP) and polio vaccine (from 86.2% to 72.3%) [5]. To understand this suboptimal vaccine uptake, a recent study showed that multiple factors, including caregiver concerns over vaccine safety and a lack of encouragement from healthcare providers [5], contributed to vaccine hesitancy. As these identified factors potentially represent different avenues for targeted public health interventions to improve vaccine uptake, this underscores the importance of disentangling and better understanding the factors underlying vaccine hesitancy.

Thus far, the COVID-19 vaccination program in FBiH has suffered a series of setbacks from an originally projected start date of late January or mid-February 2021, with the first vaccines ultimately administered towards the second week of March 2021. This delay coincided with the third wave of COVID-19 cases in the European Region [6], sparking anger and mistrust [7]. To ensure significant vaccine uptake while minimizing mistrust, behavioural insights into the FBiH population are critical. Here, we explore the attitudes that residents of the FBiH have towards COVID-19 vaccines to assess which factors should be emphasized during vaccine introduction in FBiH, as well as to shed light on the state of vaccine hesitancy in FBiH at different stages of the pandemic.

## Methods

### Survey and Study Design

Three cross-sectional surveys were conducted in 2020 (July, September, and December), before the COVID-19 vaccine was introduced in FBiH. Each involved approximately 1,000 study participants. The survey questionnaire used was adapted from a Behavioural Insights survey tool [8] developed by the World Health Organization (WHO) Regional Office for Europe, in conjunction with the University of Erfurt (see Supplementary File S1 for the full questionnaire in English). Survey participants are asked to rate their thoughts about various drivers, barriers, and attitudes regarding a potential COVID-19 vaccine on a Likert scale of 1–7, with 1 being “strongly disagree,” or “not important at all,” and 7 being “strongly agree,” or “very important.” In addition to questions regarding knowledge of the pandemic, the tool includes constructs that are more complex, such as risk perception, self-efficacy, trust, affect, fairness, prevention, resilience, worry and conspiracy thinking.

The protocol and questionnaire were reviewed by a group representing leading global experts in behavioural insights research for health and in developing and validating survey tools. Prior to implementation for this current study, the tool was validated through six rounds of data collection in Germany, translated into the local language, adapted and peer reviewed by two senior public health scientists in FBiH. The project was approved by the ethical committee of the Institute for Public Health of FBiH and by the WHO Ethical Review Committee.

### Survey Data Collection

Data were collected by a survey research company using online panels, with data collection and data delivery conducted within 72 h from survey initiation. Sampling, quota monitoring and invitational activities were performed using appropriate methodology to achieve representativeness of FBiH sample in terms of age, sex and geographical distribution. Differences in the demographic characteristics of survey participants across survey waves were analyzed using Kruskal-Wallis rank sum and Pearson’s chi-squared tests. As can be seen in Table 1, the mean age of participants was in the early 40s for all waves, ranging from 18 to 74 years of age, while there were slightly more female than male respondents. For all waves, fewer participants identified as living in a rural area (45%, 36% and 37% across waves, respectively) compared to urban (55%, 64% and 63% across waves, respectively). Regarding education, three-quarters of participants listed high school as their highest level of education completed, while over one quarter had completed college (25–27%). Across waves, approximately one-fifth of respondents were currently suffering from a chronic illness.

**TABLE 1:** Description of survey respondents (Federation of Bosnia and Herzegovina, Bosnia and Herzegovina, 2020).

Characteristic	Wave 1 (*n* = 1,000)	Wave 2 (*n* = 1,067)	Wave 3 (*n* = 1,068)	*p*-value^a^
Age				<0.001
Median (IQR)	42 (31, 55)	40 (30, 52)	45 (32, 55)	
Range	18, 74	18, 74	18, 74	
Sex				0.890
Male	483 (48%)	524 (49%)	514 (48%)	
Female	517 (52%)	543 (51%)	554 (52%)	
Area of residence				<0.001
Rural	452 (45%)	379 (36%)	398 (37%)	
Urban	548 (55%)	688 (64%)	670 (63%)	
Education				0.543
Primary or high school	730 (73%)	794 (74%)	802 (75%)	
College	270 (27%)	273 (26%)	266 (25%)	
Working in healthcare				0.783
MD	13 (1.3%)	12 (1.1%)	15 (1.4%)	
Nurse	39 (3.9%)	46 (4.3%)	42 (3.9%)	
Pharmacist	5 (0.5%)	10 (0.9%)	10 (0.9%)	
Other	26 (2.6%)	39 (3.7%)	29 (2.7%)	
Not working in healthcare	917 (92%)	960 (90%)	972 (91%)	
Suffering from chronic illness				0.061
No chronic illness	808 (81%)	872 (82%)	831 (78%)	
Chronic illness	192 (19%)	195 (18%)	237 (22%)	

a Kruskal-Wallis rank sum test; Pearson’s Chi-squared test.

### Statistical Analysis

Survey responses were first analyzed using descriptive statistics, with differences calculated using Kruskal-Wallis rank sum test and Pearson’s chi-squared test for continuous and categorical data, respectively. Following this, a fixed-effects (FE) ordinary least squares regression was fitted to the data, using responses to the statement “If a COVID-19 vaccine becomes available and is recommended for me, I would get it” as the dependent variable. The regression analysis was conducted on a pooled data set that contains all three waves of the survey. To rule out the possibility that the relationship between vaccine characteristics and acceptance is conditional upon the temporal evolution of the pandemic, we run an additional set of regressions where we add an interaction between the survey wave indicator and each question related to the importance of the vaccine based on its characteristics (e.g., Importance of the vaccine being recommended by GP). Results reported in the Supplementary Table S1 reveal that the effect of vaccine characteristics is homogenous across waves and, thus, not dependent on the dynamics of the vaccination campaign or media exposure.

FE regressions control for the serial cross-sectional study design by taking into account the across-wave variation in individual responses. This is accomplished by including an indicator for each wave of the data collection, resulting in unbiased estimates for the individual level predictors included in the model. The model was estimated without any linear transformation of the outcome and predictor variables; multicollinearity diagnostics (variance inflation factor) were performed to rule out the existence of multicollinearity among the predictors. As a robustness check, we fit an additional regression model employing robust standard errors. The standard errors are very similar across both models. Results are reported in the Supplementary Table S2. Tests for heteroskedasticity and normality of the distribution of residuals were also conducted. Complete case analyses were performed using listwise missing values deletion. All analyses were performed in R (R Core Team, 2021), using version 4.0.5.

For the complete list of variables included in the regression model, see Table 2. As the table suggests, some variables were included as averaged indices, whereby each index was calculated per respondent; only those variables that were available in each particular wave were included. The internal consistency was satisfactory for all indices, these indices are the following:

1) The perceived risk of COVID-19, which includes the respondent’s self-assessed probability of getting infected with COVID-19, his/her susceptibility to COVID-19 and the likely severity of illness if infected with COVID-19 [9];
2) Well-being, which is computed based on the WHO 5-item well-being scale (WHO-5) [10];
3) Negative affective states, which includes the respondent’s feelings of stress, helplessness, fear and depression [11];
4) Trust in sources of information, which includes trust in the Ministry of Health, trust in the Institute of Public Health and trust in health workers [12, 13].

**TABLE 2:** Explanatory factors associated with the responses to the variable “If a COVID-19 vaccine becomes available and is recommended for me, I would get it” (Federation of Bosnia and Herzegovina, Bosnia and Herzegovina, 2020).

Predictors	Estimates	CI
Intercept	1.69***	1.16, 2.23
Age: 29–38 (Ref: 18–28)	−0.20	−0.41, 0.01
Age: 39–48 (Ref: 18–28)	−0.10	−0.31, 0.12
Age: 49–58 (Ref: 18–28)	0.10	−0.12, 0.32
Age: 59–68 (Ref: 18–28)	0.05	−0.20, 0.31
Age: > 69 (Ref: 18–28)	0.47*	0.07, 0.87
Female (Ref: Male)	−0.52***	−0.66, −0.38
Urban (Ref: Rural)	0.16*	0.02, 0.30
Education: College (Ref: Primary or High School)	0.20*	0.04, 0.36
Chronically ill (Ref: No)	−0.03	−0.22, 0.16
Living alone (Ref: No)	0.09	−0.13, 0.32
Importance of the country in which the vaccine is produced	−0.18***	−0.21, −0.14
Importance of the vaccine being recommended by GP	0.01	−0.04, 0.06
Importance of the vaccine being recommended by the Ministry of Health	0.15***	0.10, 0.20
Importance of the vaccine not having serious side-effects	−0.04	−0.10, 0.02
Importance of the vaccine being used in other countries	0.19***	0.13, 0.25
Importance of the risk of getting infected when vaccine is available	−0.06*	−0.12, −0.01
Importance of the vaccine being easy to get	0.17***	0.12, 0.22
Importance of the vaccine being free of charge	0.02	−0.02, 0.05
Wellbeing	−0.02	−0.08, 0.04
Index of negative affective states (e.g., anxiety)	0.24***	0.13, 0.35
Perception of COVID-19 risk (probability, susceptibility, severity)	0.47***	0.25, 0.69
Having been infected with COVID-19 (Ref: No)	−0.04	−0.33, 0.24
Knowing peers who were infected with COVID-19 (Ref: No)	−0.09	−0.29, 0.10
Index of trust in health institutions and professionals	0.18***	0.13, 0.23
Feeling that COVID-19 is media hyped	−0.15***	−0.19, −0.12
Fixed Effect: Wave 2 (Ref: Wave 1)	0.04	−0.28, 0.36
Fixed Effect: Wave 3 (Ref: Wave 1)	0.30*	0.00, 0.60
Observations	2,964	
*R* ^2^/*R* ^2^ adjusted	0.279/0.273	

*p < 0.05, **p < 0.01, ***p < 0.001.

The Cronbach alpha coefficients reveal that selected instruments capture with a satisfactory degree of reliability the underlying concepts of respondents’ wellbeing (alpha = 0.91), trust in health institutions and professionals (alpha = 0.91), and negative affective states (alpha = 0.76). The “Perception of COVID-19 risk” index shows lower, but still acceptable, levels of internal consistency (alpha = 0.63).

## Results

**FIGURE 1:**
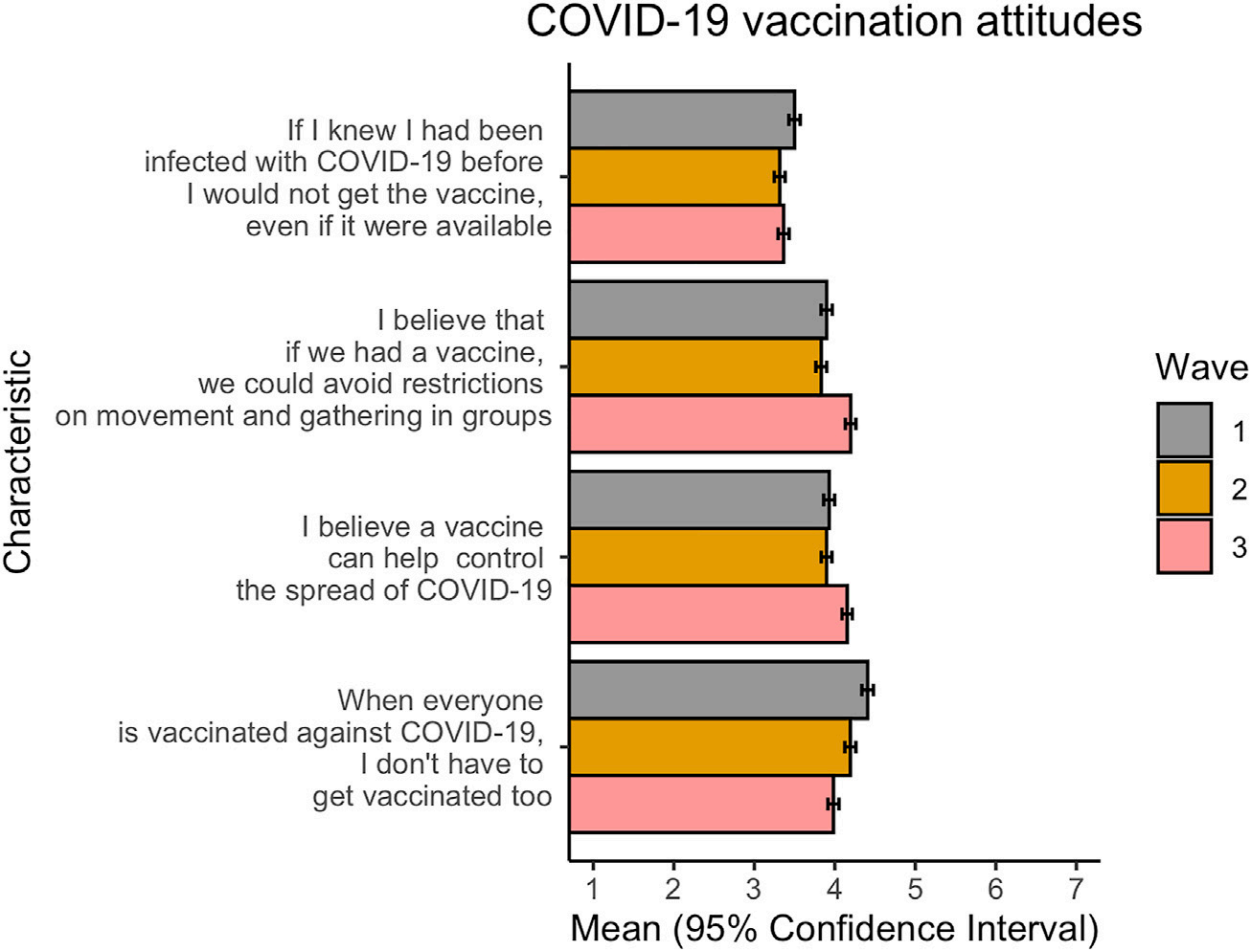
Differences in attitudes towards a COVID-19 vaccine over time. Wave 1: July 2020; Wave 2: September 2020; Wave 3: December 2020. Responses are on a 7-point scale, where 1 = strongly disagree and 7 = strongly agree (Federation of Bosnia and Herzegovina, Bosnia and Herzegovina, 2020).

Understanding attitudes towards the vaccine and towards vaccination are key behavioural insights that will be central to a successful vaccination roll-out in FBiH. The results of our surveys indicate that these variables were relatively stable over time in the study period from July to December 2020 (Figure 1), with any differences occurring in a narrow range of values on the 7-point Likert scale. Within-variable comparisons over time reveal a higher agreement with the statement, “I believe that if we had a vaccine, we could avoid restrictions on movement and gathering in groups,” in December (wave 3), compared to July and September. A similar trend is observed in response to the statement, “I believe a vaccine can help prevent the spread of COVID-19,” with a slight uptick in positive agreement in December. Conversely, there has been successively more disagreement with the statement, “When everyone is vaccinated against COVID-19, I don’t have to get vaccinated too,” across all three waves. This is echoed by slightly more disagreement regarding the statement, “If I know I had been infected with COVID-19 before, I would not get the vaccine even if it were available,” from July and September.

**FIGURE 2:**
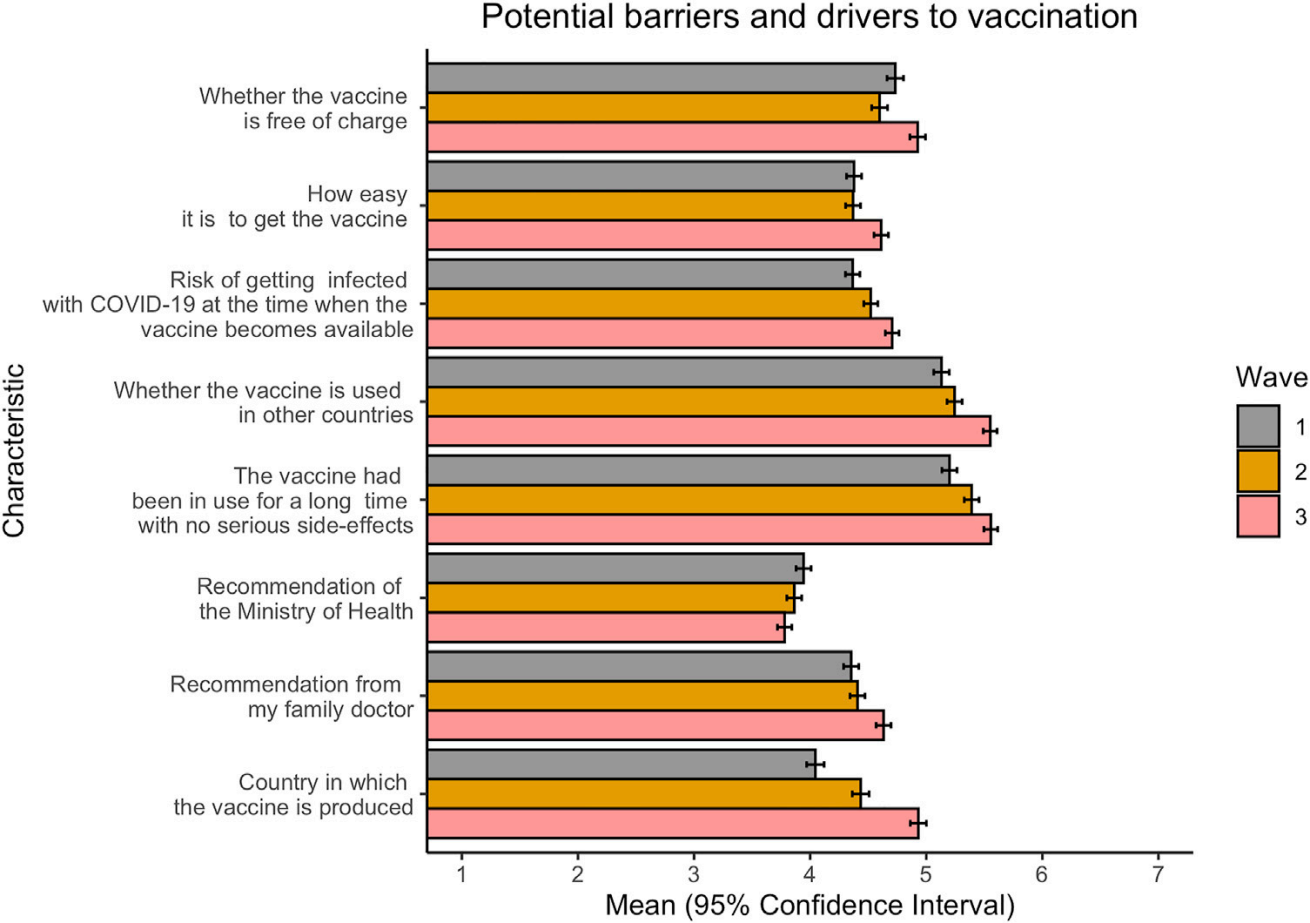
Differences in potential barriers and drivers to COVID-19 vaccination over time. Wave 1: July 2020; Wave 2: September 2020; Wave 3: December 2020. Responses are on a 7-point scale, where 1 = strongly disagree and 7 = strongly agree (Federation of Bosnia and Herzegovina, Bosnia and Herzegovina, 2020).

To determine which factors could be potential drivers—or the reverse, barriers—to vaccination, survey respondents were asked to rate their agreement with several variables in regard to vaccination, using a 7-point scale. As compared to our survey questions on COVID-19 vaccination attitudes, a greater variation in responses, both between variables and within-variables over time, was observed regarding potential barriers and drivers to vaccination (Figure 2). Overall, whether the vaccine was used in other countries and whether the vaccine had been in use for a long-time with no serious side-effects had the highest positive responses across all waves. This indicates that these variables, which both relate to vaccine safety, were the most important drivers to vaccination in the survey populations during the period from July to December, consistently outranking other putative drivers to vaccination, including ease and cost of obtaining the vaccine, current risk of COVID-19 infection at the time of vaccine roll-out and recommendations from a family doctor. A recommendation from the Ministry of Health, on the other hand, scored slightly negative scores that were relatively consistent over time, indicating that this variable is likely not a driver of vaccination in the population.

**FIGURE 3:**
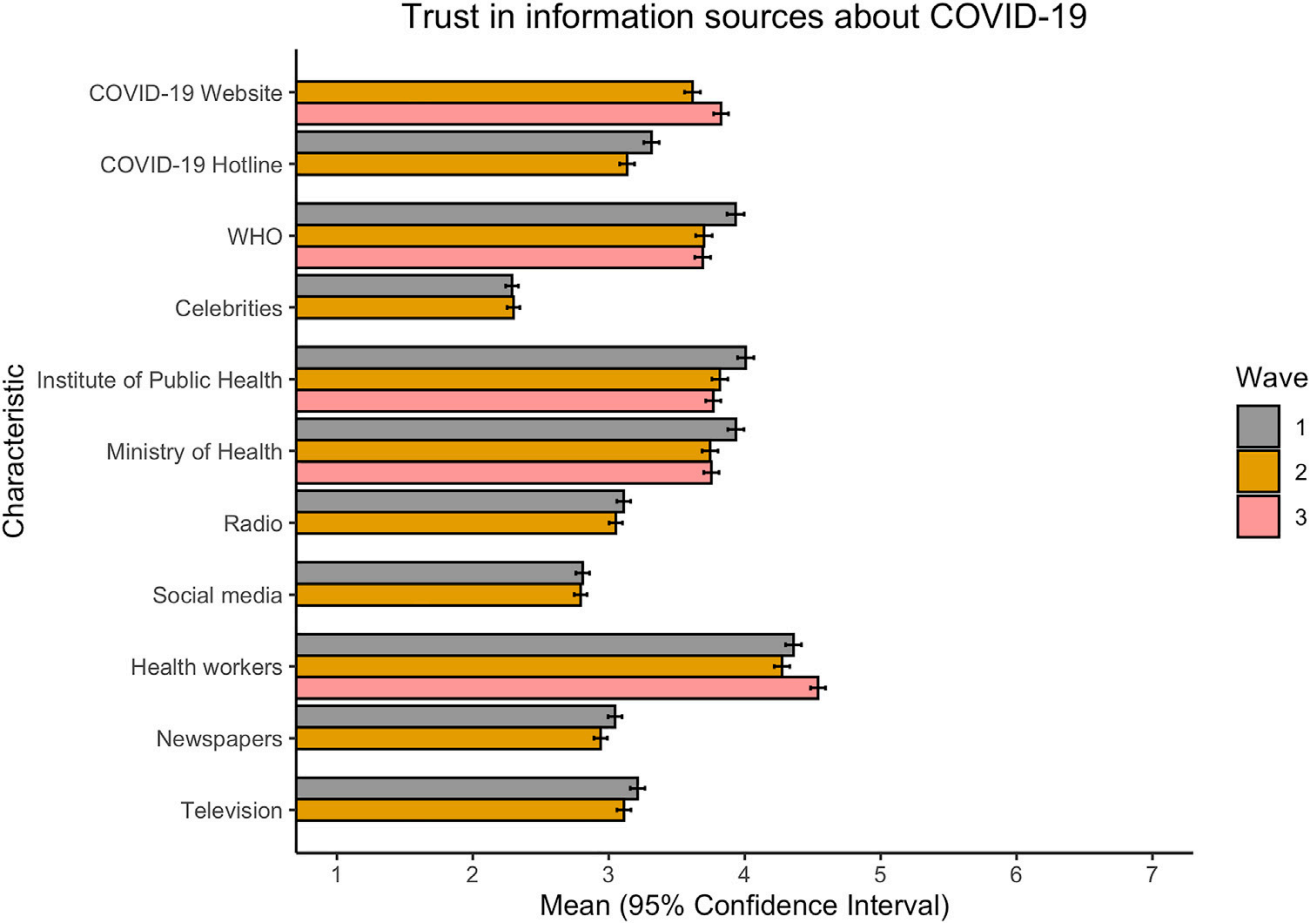
Differences in trust in information sources about COVID-19 vaccine over time. Wave 1: July 2020; Wave 2: September 2020; Wave 3: December 2020. Responses are on a 7-point scale, where 1 = strongly disagree and 7 = strongly agree. NB: not all variables were collected in Wave 3 (Federation of Bosnia and Herzegovina, Bosnia and Herzegovina, 2020).

High trust in information sources regarding COVID-19 underlies a successful vaccination campaign, yet trust varies both between and within countries over time. To understand which information sources were most highly trusted in FBiH and whether this changed over time, respondents were asked to rate their agreement regarding trust for each variable. From Figure 3, it can be seen that while there are some observable within-variable differences over time, the greatest variation occurred between information sources, with social media and celebrities consistently being rated as the least trustworthy (mean values between 2 and 3) and health workers rated the most trustworthy (mean values between 4 and 5). As any mean value above 4 indicates that respondents rate the source as trustworthy, it is worthwhile to consider that only health workers are considered trustworthy, and that only slightly so. Mean responses regarding the federal COVID-19 website, WHO, the Institute of Public Health, and the Ministry of Health cluster at values slightly less than 4 on the Likert scale, indicating neutral to slightly untrustworthy views on these information sources with regards to COVID-19.

**FIGURE 4:**
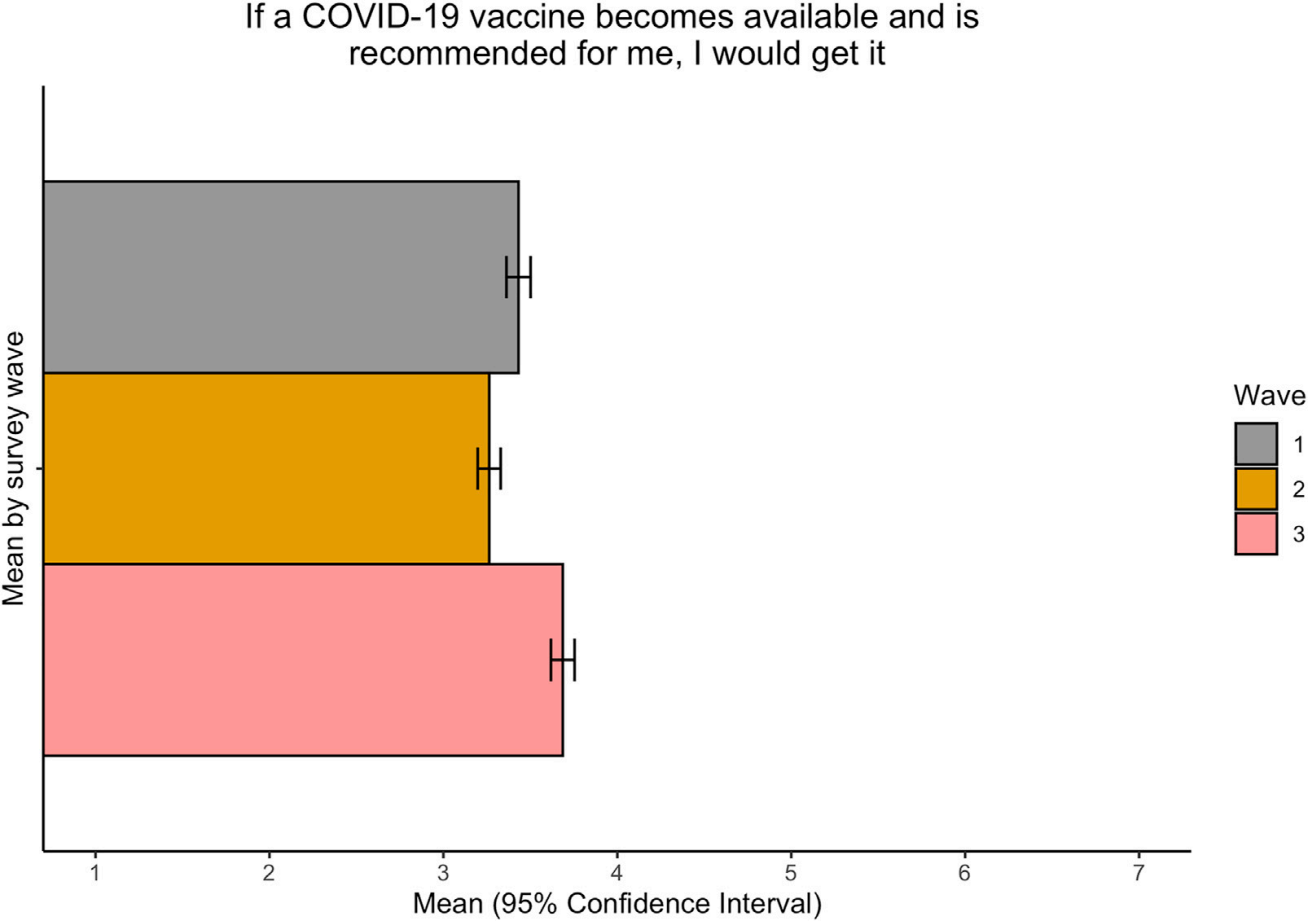
Average responses to the question “If the vaccine becomes available and is recommended for me, I would get it” over time. Wave 1: July 2020; Wave 2: September 2020; Wave 3: December 2020. Responses are on a 7-point scale, where 1 = strongly disagree and 7 = strongly agree (Federation of Bosnia and Herzegovina, Bosnia and Herzegovina, 2020).

At each wave of the survey, respondents were asked to rate their response to the statement “If a COVID-19 vaccine becomes available and is recommended for me, I would get it,” again using the Likert 7-point scale. When pooled across survey waves, the share of respondents answering negatively to this question—in other words, they would not get the vaccine even if it were recommended to them—is greater than those who would get the vaccine (Supplementary Figure S1). Calculating the average value of ratings for each wave shows that both the mean values and the 95% confidence intervals fall between the values of 3 and 4, indicating that the majority of respondents would choose to not get the vaccine (Figure 4). There is, however, temporal variation within this, with respondents rating this statement less negatively in December than in previous survey waves conducted in the summer and autumn months.

To explore which demographic, trust, attitudinal and wellbeing variables might affect vaccine hesitancy, a regression analysis was performed using responses to the statement “If a COVID-19 vaccine becomes available and is recommended for me, I would get it” as the dependent variable. Being older (age over 69, *β* = 0.47) and having a perception of COVID-19 risk (*β* = 0.47) were statistically significant predictors of a positive response—choosing to get the vaccine—as can be seen from Table 2. To interpret the results of a fixed effects regression, an estimate of +0.47 indicates that this variable contributes this same amount to the response observed on the Likert 7-point scale. Additional significant predictors of a positive effect were having a negative affective state, having a college education, trusting health professionals and institutions, the vaccine being recommended by the Ministry of Health, the vaccine being used in other countries and the vaccine being easy to obtain, albeit to a lesser extent. In the other direction—choosing to not get the vaccine—being female had the highest effect on the response (*β* = −0.52), while country where the vaccine was produced, a feeling that COVID-19 was hyped by the media and risk of getting infected from COVID-19 were all significant predictors, although to a lesser degree.

## Discussion

Vaccine hesitancy has been attributed to vaccine safety concerns, lack of knowledge about vaccination, the acceptability and convenience related to vaccine service provision and socioeconomic, religious, and cultural issues surrounding vaccine [14]. Given that it is highly contextual to place and time, this necessitates conducting granular-level analyses on a country-by-country basis. Using a snapshot approach, we provided the first insights into factors affecting willingness to accept the COVID-19 vaccine in FBiH before it was even introduced.

Our findings demonstrated that healthcare workers were consistently considered to be the most trustworthy source of information. When we incorporated an index of trust in health institutions and professionals, which includes trust in the Ministry of Health, trust in the Institute of Public Health and trust in health workers, in the regression on the variable “If a COVID-19 vaccine becomes available and is recommended for me, I would get it,” trust emerged as a highly significant predictor of positive vaccine intentions. This corroborates recent research assessing COVID-19 vaccine acceptance in 19 countries, whereby respondents who reported a higher trust in government were more likely to get accept a COVID-19 vaccine [15], as well as findings from neighbouring Serbia [16]. Prior to COVID-19, it was recognized that trust factored strongly on the level of vaccine uptake in immunization initiatives. For example, during the polio eradication initiatives in the Democratic Republic of Congo, a deep sense of distrust in government health services increased vaccine avoidance behavior, and subsequently weakened the impact of eradication efforts [17]. Mistrust in governments can be caused by numerous factors, ranging from pharmaceutical industry controversies as in the case of France [18], to more extreme factors such as betrayal of trust in previous immunization initiatives as in the case of Pakistan [19].

The index of risk perception, which includes the respondent’s self-assessed probability of getting infected with COVID-19, his/her susceptibility to COVID-19 and the likely severity of illness if infected with COVID-19, was a highly significant driver of positive vaccine intentions in this survey. Certain risk factors such as older age, underlying medical conditions and working in a profession such as healthcare which may require close contact with COVID-19 positive patients can all increase risk [20]. Additionally, the pace at which vaccination is rolled out, combined with the stringency of the presence of non-pharmaceutical interventions such as curfews, and masking mandates, affect transmission dynamics of the virus, and therefore affect one’s risk of becoming infected. While getting vaccinated does reduce the risk of transmission of the virus and substantially reduces the risk of becoming seriously infected with SARS-CoV-2, there is still a risk of infection, including when an individual is in-between doses for vaccines that require two doses, as well as for approximately two weeks after getting vaccinated [21, 22]. Another factor to consider is individuals who have already been infected with the SARS-CoV-2 virus. The mean value for the attitude “If I knew I had been infected with COVID-19 before, I would not get a vaccine, even if it were available” was consistently low between survey waves. Research from Italy suggests that being hospitalized for COVID-19 was not associated with willingness to accept the COVID-19 vaccine, indicating that those who recover from COVID-19 also have complex hesitancy views [23]. While previously infected individuals will have immunity for some time, this will eventually begin to wane [24]. Clarifying these misconceptions through messaging is crucial for achieving satisfactory vaccine uptake and reducing viral transmission.

Safety concerns were the most important variables, with “whether the vaccine is used in other countries” and “whether the vaccine has been in use for a long time with no side-effects” consistently being scored highest among the factors influencing a decision to get vaccinated. Historically, concerns over vaccine hesitancy have led to significant drops in vaccination coverage in otherwise strong vaccination programmes [25], and recent studies have shown that vaccine safety is a critical factor for vaccine hesitancy in FBiH [4, 5].

Having negative emotional affect as a reaction to the pandemic, which was a composite score of feelings of stress, helplessness, fear and depression in our analysis, strongly predicted positive vaccine intentions. This could be reflective of the reality that those suffering from severe mental illness more likely bear an increased risk of COVID-19 infection, as well as COVID-19 related morbidity and mortality, and have therefore been recommended for prioritization in vaccine allocation strategies [26]. This link between negative affect and positive vaccine intentions could also reflect a greater awareness of the severity of the disease, as was recently shown in a Finnish sample [27]. Alternatively, negative affect might drive vaccine intentions due to worry about loved ones contracting the disease, as was found in a study on COVID-19 vaccine attitudes in Turkey [28].

Being female was a highly significant negative predictor on positive vaccine intentions (Table 2) despite the fact that the same surveys in FBiH do not indicate any lower risk perception or adherence to other preventive measures in women than in men. This finding substantiates a study over the same time period from Turkey [28], where being female was a significant factor in vaccine refusal, as well as from the United States [29]. Given the average age of respondents, this could reflect concerns regarding the impact of the vaccine on pregnancy or fertility. Indeed, recent research from Qatar, where perinatal women exhibited a vaccine hesitancy rate of 25% towards COVID-19 immunization, cited as their main concerns infection risks and vaccine safety [30]. Women, too, have stressed potential impacts to fertility as barriers to vaccine acceptance [31], as well as living with children [32]. Further exploring the specific concerns of women in the FBiH would illuminate what is driving their hesitancy to accept a vaccine.

The country in which the vaccine was produced had opposite effects on the dependent variable. These trends align with other surveys assessing COVID-19 vaccine acceptance. In a survey conducted in Brazil, for instance, participants were asked how likely they were to receive a COVID-19 vaccine with and without mention of a country of origin of the vaccine [33]. When a country of origin was not specified, 88.3% reported being either likely or very likely to get vaccinated, whereas when a country of origin was specified as either China or the Russian Federation, positive vaccine intentions decreased to only 67.0% and 72.6% (respectively) [33]. Approval of vaccines by large regulatory bodies such as the WHO has been shown to increase vaccine confidence [6]. However, the administration of vaccines to the general public prior to the start of phase 3 clinical trials, as in the case of some vaccines, has been speculated to heighten vaccine hesitancy [6]. Circumstances have dictated that FBiH had to rely on a variety of vaccines in order to have enough vaccines to cover the population of FBiH.

Access to vaccines is another important component of vaccine hesitancy and acceptance [34]. Questions related to access in this survey included “Whether the vaccine is free of charge” and “How easy it is to get the vaccine” both of which became an increasingly important driver of positive vaccine intentions from the second to the third wave. This substantiates previous studies in FBiH that have indicated that ease of access is an important factor for vaccination uptake [4, 5, 35]. A survey assessing COVID-19 vaccine hesitancy of a working-age population in France further corroborates this trend, and suggests that vaccine hesitancy in their study population increased if vaccines were only available in mass vaccination clinics, rather than doctor’s offices, or pharmacies [18]. Previous issues with accessibility of other vaccines may also influence an individual’s perception of access to a COVID-19 vaccine [36]. In a survey conducted in Chile, researchers found that many respondents are willing to pay for a COVID-19 vaccine [37]. However, when the same respondents were asked if they would be willing to pay for a COVID-19 vaccine at a higher price than what was stated in the original question, 12% stated that they would not be willing to pay [37]. COVID-19 vaccines are free of charge and are recommended to FBiH residents.

There are several limitations to this study. For context, the first wave of the survey was conducted in July 2020; prior to this time, some vaccine candidates were in the midst of completing Phase 1 or Phase 2 clinical trials [33]. Other vaccine candidates, such as the mRNA-based Moderna, and the Pfizer/BioNTech vaccines, proceeded towards the Phase 3 clinical trial stage, where thousands of volunteers around the world would be enrolled [6]. Uncertainty still loomed in the general public around this time, as both of the front running vaccines utilize technology that has never been used previously. The second wave of the study was conducted in September 2020, closely following the announcement that the Russian Sputnik V vaccine had become the first vaccine for SARS-CoV-2 in the world. Also, during this time period, the AstraZeneca vaccine trial was briefly paused due to safety concerns, sparked by a trial participant who experienced an adverse reaction to the vaccine. This may have caused concerns about the safety of vaccines, and thus increased feelings of hesitancy [37]. The third wave of the survey occurred in December, when the United Kingdom became the first country in the world to begin administrating the Pfizer/BioNTech vaccine [29]. Following this, several other vaccines received emergency use authorizations, and began rolling out around the world in December [6, 33]. While the aim of the study was to provide temporal snapshots of changing attitudes and perceptions, the fact that it was conducted at a time when vaccines were not available in FBiH might affect generalizability over time.

Second, given that the survey was delivered online, individuals with limited internet access may have not been able to participate, which might explain the greater proportion of urban respondents. Third, even if quota sampling is used to ensure as representative a sample as possible, some population groups are expected not to be reached, including disadvantaged population groups such as migrants, homeless people, people with some mental health conditions: those who may very well be more affected by the pandemic than the average citizen in the FBiH [38]. As the survey cannot be claimed to represent their views, the social benefit of the study may consequently be reduced. Conducting more tailored and targeted surveys with specific population groups would serve to help rectify this inequity. Fourth, given the complexity of the pandemic and the response to it, this survey can only identify issues of concern that should ultimately be complemented by qualitative interviews that can better provide contextual information. Last, these surveys were completed before the actual introduction of any vaccine which may have affected answers. Additionally, it is well-known that there can be a considerable gap between intentions and behaviour, and thus the positive and negative vaccine intentions explored in this paper may not be translated directly into positive or negative vaccine behaviours by the respondents.

To summarize, important insights were gained that have informed the continued COVID-19 vaccine introduction strategy in FBiH. Overall, trust was found to be a driver for positive vaccine intentions, and health workers are among the most trusted, proving the importance of supporting, training and motivating health workers as part of the vaccine introduction strategy. Likewise, the perceived risk of the coronavirus was found to drive positive vaccine intentions and thus continued strategies to ensure appropriate knowledge and risk perceptions in the population are key. The fact that women and those with lower than college education were found to be less likely to accept the vaccine warrant targeted strategies for these groups, again with health workers as trusted mediators. The complexity of factors influencing vaccine intentions demonstrate that vaccine hesitancy cannot be addressed by health institutions and medical doctors alone. Rather, a joint effort among other stakeholders, governmental and non-governmental organizations, academia, and the general public, is required to make an impact. To unify these groups, there must be clear, direct communication between one another [39]. These stakeholders all play varying roles in regard to strengthening positive social norms for vaccination, sharing correct information and dispelling vaccination misconceptions, and strengthening vaccination services and outreach and have varying audiences to which they appeal to the most. By fostering successful collaboration between these entities, public trust and vaccine confidence will increase, and efforts to increase vaccine acceptance and uptake can have a much greater reach [39, 40].
