# Supplementary material for: COVID-19 Risk Perception, Trust in Institutions and Negative Affect Drive Positive COVID-19 Vaccine Intentions
Source: Int J Public Health. 2022 Apr 11;67:1604231. doi: 10.3389/ijph.2022.1604231 (PMC9036943; doi:10.3389/ijph.2022.1604231)

## Supplementary File 2

## Appendix: COVID-19 risk perception, trust in institutions and negative affect drive positive COVID-19 vaccine intentions

### Homogeneity of the effect of the importance of vaccine characteristics across survey waves

To rule out the possibility that the relationship between vaccine characteristics and acceptance is conditional upon the temporal evolution of the pandemic, we run an additional set of regressions where we add an interaction between the survey wave indicator and each question related to the importance of the vaccine based on its characteristics (e.g. Importance of the vaccine being recommended by GP). Results reveal that the effect of vaccine characteristics is homogenous across waves and, thus, not dependent on the dynamics of the vaccination campaign or media exposure. The only exception is the relationship between the country of production of the vaccine and acceptance in Wave 3. Despite the beta coefficient between the country of production is relatively small (0.09), this result suggests an increasing awareness of the respondents that might be related to the controversies over Russian and Chines vaccines. Due to space constraints, in the Appendix, we report a single regression model with all the interactions fitted at once.

| **Supplementary Table S1.** Predictors of responses to the variable “If a COVID-19 vaccine becomes available and is recommended for me, I would get it” including interaction effects between importance of vaccine characteristics and survey waves (Federation of Bosnia and Herzegovina, Bosnia and Herzegovina, 2020). | | |
| --- | --- | --- |
| **Predictors** | **Estimates** | **CI** |
| Intercept | 1.81 ^***^ | 1.19 , 2.44 |
| Age: 29-38 (Ref: 18-28) | -0.19 | -0.40 , 0.02 |
| Age: 39-48 (Ref: 18-28) | -0.09 | -0.31 , 0.12 |
| Age: 49-58 (Ref: 18-28) | 0.10 | -0.12 , 0.32 |
| Age: 59-68 (Ref: 18-28) | 0.07 | -0.19 , 0.33 |
| Age: > 69 (Ref: 18-28) | 0.46 ^*^ | 0.06 , 0.86 |
| Female (Ref: Male) | -0.54 ^***^ | -0.67 , -0.40 |
| Urban (Ref: Rural) | 0.16 ^*^ | 0.02 , 0.30 |
| Education: College (Ref: Primary or Highschool) | 0.20 ^*^ | 0.05 , 0.36 |
| Chronically ill (Ref: No) | -0.03 | -0.21 , 0.16 |
| Living alone (Ref: No) | 0.09 | -0.14 , 0.32 |
| Importance of the country in which the vaccine is produced | -0.23 ^***^ | -0.29 , -0.17 |
| Importance of the vaccine being recommended by GP | 0.06 | -0.54 , 0.66 |
| Importance of the vaccine being recommended by the Ministry of Health | 0.00 | -0.62 , 0.63 |
| Importance of the vaccine not having serious side-effects | 0.07 | -0.02 , 0.17 |
| Importance of the vaccine being used in other countries | 0.12 ^*^ | 0.02 , 0.21 |
| Importance of the risk of getting infected when vaccine is available | -0.03 | -0.14 , 0.08 |
| Importance of the vaccine being easy to get | 0.24 ^***^ | 0.12 , 0.36 |
| Importance of the vaccine being free of charge | -0.11 ^*^ | -0.21 , -0.00 |
| Wellbeing | 0.16 ^**^ | 0.06 , 0.26 |
| Index of negative affective states (e.g. anxiety) | 0.01 | -0.06 , 0.08 |
| Perception of COVID-19 risk (probability, susceptibility, severity) | -0.03 | -0.09 , 0.04 |
| Having been infected with COVID-19 | 0.24 ^***^ | 0.13 , 0.35 |
| Knowing peers who were infected with COVID-19 | 0.48 ^***^ | 0.26 , 0.70 |
| Index of trust in health institutions and professionals | -0.03 | -0.31 , 0.26 |
| Feeling that COVID-19 is media hyped | -0.09 | -0.29 , 0.10 |
| Fixed Effect: Wave2 (Ref: Wave 1) | 0.18 ^***^ | 0.13 , 0.23 |
| Fixed Effect: Wave3 (Ref: Wave 1) | -0.15 ^***^ | -0.19 , -0.12 |
| Importance of the country in which the vaccine is produced X Wave 2 (Ref:1) | 0.05 | -0.03 , 0.14 |
| Importance of the country in which the vaccine is produced X Wave 3 (Ref:1) | 0.09 ^*^ | 0.01 , 0.18 |
| Importance of the vaccine being recommended by GP X Wave 2 (Ref:1) | -0.13 | -0.25 , 0.00 |
| Importance of the vaccine being recommended by GP X Wave 3 (Ref:1) | -0.04 | -0.16 , 0.09 |
| Importance of the vaccine being recommended by the Ministry of Health X Wave 2 (Ref:1) | 0.05 | -0.07 , 0.18 |
| Importance of the vaccine being recommended by the Ministry of Health X Wave 3 (Ref:1) | 0.06 | -0.06 , 0.18 |
| Importance of the vaccine not having serious side-effects X Wave 2 (Ref:1) | -0.03 | -0.17 , 0.11 |
| Importance of the vaccine not having serious side-effects X Wave 3 (Ref:1) | 0.01 | -0.14 , 0.16 |
| Importance of the vaccine being used in other countries X Wave 2 (Ref:1) | -0.04 | -0.20 , 0.11 |
| Importance of the vaccine being used in other countries X Wave 3 (Ref:1) | -0.09 | -0.25 , 0.07 |
| Importance of the risk of getting infected when vaccine is available X Wave 2 (Ref:1) | 0.08 | -0.06 , 0.22 |
| Importance of the risk of getting infected when vaccine is available X Wave 3 (Ref:1) | 0.03 | -0.10 , 0.17 |
| Importance of the vaccine being easy to get X Wave 2 (Ref:1) | -0.02 | -0.15 , 0.11 |
| Importance of the vaccine being easy to get X Wave 3 (Ref:1) | 0.05 | -0.08 , 0.18 |
| Importance of the vaccine being free of charge X Wave 2 (Ref:1) | 0.05 | -0.05 , 0.14 |
| Importance of the vaccine being free of charge X Wave 3 (Ref:1) | -0.04 | -0.13 , 0.06 |
| Observations | 2964 | |
| R^2^ / R^2^ adjusted | 0.284 / 0.274 | |
| ** p<0.05   ** p<0.01   *** p<0.001* | | |

### OLS with robust standard errors

The residual variance of the model reported in the manuscript, despite being symmetric, is not uniformly distributed across the entire range of values of the outcome of interest. Given the rather weak non-uniformity, we do not expect significant differences in the estimated standard error. However, as commonly done, we fit an additional regression model to obtain unbiased standard errors of OLS coefficients under heteroscedasticity. As expected, the standard errors are very similar across both models with no significant differences in the magnitude of any of the included predictors.

| **Supplementary Table S2.** Predictors of responses to the variable “If a COVID-19 vaccine becomes available and is recommended for me, I would get it” with robust standard errors (Federation of Bosnia and Herzegovina, Bosnia and Herzegovina, 2020). | | |
| --- | --- | --- |
| ***Predictors*** | ***Estimates*** | ***CI*** |
| Intercept | 1.69 ^***^ | 1.14 , 2.25 |
| Age: 29-38 (Ref: 18-28) | -0.20 | -0.41 , 0.01 |
| Age: 39-48 (Ref: 18-28) | -0.10 | -0.31 , 0.11 |
| Age: 49-58 (Ref: 18-28) | 0.10 | -0.12 , 0.32 |
| Age: 59-68 (Ref: 18-28) | 0.05 | -0.20 , 0.31 |
| Age: > 69 (Ref: 18-28) | 0.47 ^*^ | 0.08 , 0.85 |
| Female (Ref: Male) | -0.52 ^***^ | -0.66 , -0.38 |
| Urban (Ref: Rural) | 0.16 ^*^ | 0.02 , 0.30 |
| Education: College (Ref: Primary or Highschool) | 0.20 ^*^ | 0.05 , 0.35 |
| Chronically ill (Ref: No) | -0.03 | -0.22 , 0.16 |
| Living alone (Ref: No) | 0.09 | -0.13 , 0.32 |
| Importance of the country in which the vaccine is produced | -0.18 ^***^ | -0.21 , -0.14 |
| Importance of the vaccine being recommended by GP | 0.01 | -0.04 , 0.06 |
| Importance of the vaccine being recommended by the Ministry of Health | 0.15 ^***^ | 0.10 , 0.21 |
| Importance of the vaccine not having serious side-effects | -0.04 | -0.09 , 0.01 |
| Importance of the vaccine being used in other countries | 0.19 ^***^ | 0.13 , 0.25 |
| Importance of the risk of getting infected when vaccine is available | -0.06 ^*^ | -0.12 , -0.00 |
| Importance of the vaccine being easy to get | 0.17 ^***^ | 0.11 , 0.23 |
| Importance of the vaccine being free of charge | 0.02 | -0.02 , 0.06 |
| Wellbeing | -0.02 | -0.09 , 0.05 |
| Index of negative affective states (e.g. anxiety) | 0.24 ^***^ | 0.13 , 0.35 |
| Perception of COVID-19 risk (probability, susceptibility, severity) | 0.47 ^***^ | 0.25 , 0.70 |
| Having been infected with COVID-19 | -0.04 | -0.35 , 0.26 |
| Knowing peers who were infected with COVID-19 | -0.09 | -0.29 , 0.10 |
| Index of trust in health institutions and professionals | 0.18 ^***^ | 0.13 , 0.23 |
| Feeling that COVID-19 is media hyped | -0.15 ^***^ | -0.19 , -0.11 |
| Fixed Effect: Wave2 (Ref: Wave 1) | 0.04 | -0.30 , 0.38 |
| Fixed Effect: Wave3 (Ref: Wave 1) | 0.30 | -0.02 , 0.62 |
| Observations | 2964 | |
| R^2^ / R^2^ adjusted | 0.279 / 0.273 | |
| ** p<0.05   ** p<0.01   *** p<0.001* | | |

### Distribution of the dependent variable

We plot the distribution of the dependent variable “If a COVID-19 vaccine becomes available and is recommended for me, I would get it” in Figure S3, pooling the data across waves. The share of respondents answered negatively to this question.

**Supplementary Figure S1.** Distribution of responses to the question “If the vaccine becomes available and is recommended for me, I would get it “. Data were pooled across waves. Responses are on a 7-point scale, where 1=strongly disagree and 7=strongly agree (Federation of Bosnia and Herzegovina, Bosnia and Herzegovina, 2020).


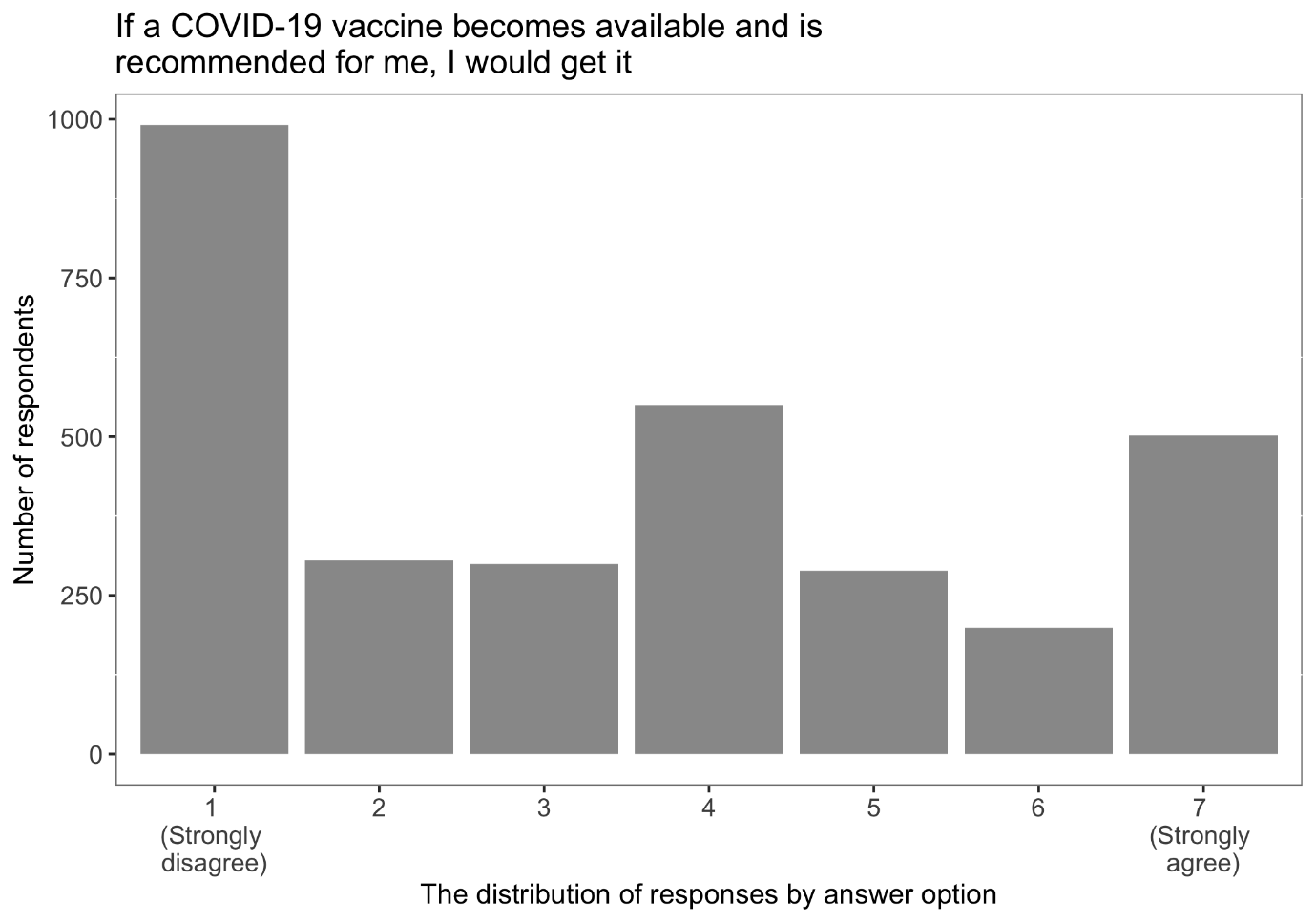

Supplement: Supplementary file 1 [file DataSheet2.docx]
