# Supplementary material for: COVID-19 Risk Perception, Trust in Institutions and Negative Affect Drive Positive COVID-19 Vaccine Intentions
Source: Int J Public Health. 2022 Apr 11;67:1604231. doi: 10.3389/ijph.2022.1604231 (PMC9036943; doi:10.3389/ijph.2022.1604231)
Supplement: Supplementary file 2 [file DataSheet1.docx]

## Supplementary File 1

| Survey questionnaire (English translation) (Federation of Bosnia and Herzegovina, Bosnia and Herzegovina, 2020). | |
| --- | --- |
| Page 1  Introduction | Dear Participant,  Thank you for taking part in this study to help improve actions taken in response to the novel coronavirus pandemic (Covid-19) and to inform the response to similar future outbreaks.  This study will involve answering a 15-minute survey which will be asking you questions relating to the coronavirus. Please do not start until you will have enough time to complete it in one go. Please close other programmes (e.g. chat or e-mail) to avoid distractions. By taking part, you are agreeing that you have read and understood the information about the study below. Please ensure you have read and understood this information before continuing.    **What is this project about, and do I have to take part?**  This study aims to inform governmental outbreak response measures, including policies, interventions and communications. The information collected through this survey is important to support the implementation of specific programmatic interventions and policies in addition to the messaging necessary to encourage uptake of those measures. Participation is open to people at the age of 18 or over, living in Federation of BiH and is entirely voluntary. You do not have to be in isolation to take part.    **What are the benefits and risks of taking part?**  You may benefit from taking part in the survey by being motivated to look up information about the coronavirus pandemic. We will provide you with good resources at the end of the study. There are no foreseeable risks for you when taking part in the survey other than time spent on the survey and potential discomfort. Should you feel uncomfortable and want to leave the study you are free to do so without any consequences.    **What will you ask and what will happen to the information I give you?**  You will be asked questions about yourself, your knowledge of the coronavirus, the actions you have taken to protect yourself from the virus, your trust in various stakeholders, and your own fears and worries relating to the coronavirus pandemic. Some of these questions are considered sensitive data, such as questions relating to your trust in your government. However, you will not be asked to provide any personal data. Your anonymous data will be collected by Valicon and analyzed by Institute of Public Health Your data will be shared, but only with relevant researchers and government agencies. However, your data will be completely anonymous, and it will not be possible to identify you individually from your answers. This study has received approval from the WHO Research Ethics Review Committee and from Ethic Committee from Institute of Public Health.  The information that is required to be provided to participants under data protection legislation (GDPR) is provided across both the 'local' and 'general' privacy notices.  The lawful bases used in this survey are that it is undertaken as a task in the public interest and necessary for research and public health purposes, in accordance with the General Data Protection Regulation and national laws.    **Consent**  I understand that:   - My participation is completely voluntary. - All my answers will be used for scientific research to improve actions taken in response to the coronavirus pandemic and to inform the response to similar future outbreaks. - My data will be stored securely, however, no personal data will be stored, and my answer will be completely anonymous. - My data gathered in this study will be shared with relevant researchers and government agencies. - Because I am submitting anonymous data, it will not be possible to withdraw my answers after they have been submitted.     Please note that you can stop the survey at any time. This will not entail any penalty, and it will not affect the services (health care services or others) that you receive.  By ticking the box, you are agreeing that you are at least 18 years old, that you have read the information about the study, and that you voluntarily agree to take part in it.  [*] I agree to participate in this study. |
| Page 2    Variable:  socio-demography    [Screen out: <18] | **How old are you?**  I am ____ years old.  **What is your gender?**  [*] Male  [*] Female  [*] Other  **How many years of education have you completed?**  [*] Primary school  [*] High school  [*] College  **Are you a medical professional?**  [*] Nurse  [*] Medical doctor  [*] Pharmacist  [*] Other  **Do you have a chronic illness?**  [*] Yes  [*] No  [*] Don’t know  **How many** inhabitants **live in the village or town in which you live?**  [*] ≤ 5,000 inhabitants  [*] 5,001 - 20,000 inhabitants  [*] 20,001 - 100,000 inhabitants  [*] 100,001 - 500,000 inhabitants  [*] > 500,000 inhabitants  [*] Do not know  **Where do you live?**  [*] Rural area  [*] Urban area  **In which Canton do you live?**  [*]Una-Sana Canton (267,874)  [*] Posavina Canton (41,346)  [*] Tuzla Canton (438,811)  [*] Zenica-Doboj Canton (358,292)  [*] Bosnia-Podrinje Canton (23,041)  [*] Central Bosnia Canton (249,879)  [*] Herzegovina-Neretva Canton (216,970)  [*] West Herzegovina Canton (93,385)  [*] Sarajevo Canton (420,496)  [*] Canton 10 (80,004)  **Do you have children living at home with you?**  [*] Yes [*] No  **How many people live in your household, including yourself?**  [*] I live alone [*] I live with 1 or more persons |
| Page 3  Variable: Risk group  [Single choice]        [Single choice]    [Multiple choice for yes] | **Are you, or have you been, infected with the novel coronavirus?**  [*] Yes, tested and the result was positive  [*] Yes, suspected but not confirmed by a test  [*] No, tested and the result was negative  [*] No [*] Don't know  **Do you know people in your immediate social environment who are or have been infected with the novel coronavirus?**  [*] Yes, confirmed  [*] Yes, suspected but not confirmed by a test  [*] No, tested and the result was negative  [*] No [*] Don't know  Please click CONTINUE to proceed |
| Page 4  Variable: Self-assessed knowledge | **How would you rate your knowledge level on how to prevent spread of the novel coronavirus?**Very poor knowledge [*] [*] [*] [*] [*] [*] [*] Very good knowledge  Please click CONTINUE to proceed |
| Page 5  Variable: Knowledge symptoms, treatment      [Random order of symptom items]                [Random order of the first two answer options] | **Which of the following can be symptoms of the novel coronavirus?**Please select as many as apply  **Fever**  [*] Related to the newly emerged coronavirus  [*] Not related to the newly emerged coronavirus  [*] Don’t know (same response categories for each of the symptoms listed below)  **Cough**[Answer scheme: see “Fever”]  **Shortness of breath**[Answer scheme: see “Fever”]  **Sore throat**[Answer scheme: see “Fever”]  **Runny or stuffy nose**[Answer scheme: see “Fever”]  **Muscle or body aches**[Answer scheme: see “Fever”]  **Headaches**[Answer scheme: see “Fever”]  **Fatigue (tiredness)**[Answer scheme: see “Fever”]  **Diarrhea**[Answer scheme: see “Fever”]  **Loss of taste and smell** [Answer scheme: see “Fever”]  **Which answer is correct?**  [*] There is a drug to treat the novel coronavirus.  [*] There is a vaccine for the novel coronavirus.  [*] There is both a drug for the treatment and a vaccine for the novel coronavirus.  [*] There is currently no drug treatment or vaccine for the novel coronavirus.  [*] Don’t know  Please click CONTINUE to proceed |
| Page 6  Variable: Knowledge incubation | **What is the** maximum **incubation period (i.e., the time from viral infection to developing symptoms of illness) of the novel coronavirus?**  [*] Up to 3 days [*] Up to 7 days [*] Up to 14 days [*] Don’t know  Please click CONTINUE to proceed |
| Page 7  Variable: Probability and Severity | **What do you consider to be your own probability of getting infected with the novel coronavirus?**Extremely unlikely [*] [*] [*] [*] [*] [*] [*] Extremely likely  **How susceptible do you consider yourself to an infection with the novel coronavirus?**Not at all susceptible [*] [*] [*] [*] [*] [*] [*] Very susceptible  **How severe would contracting the novel coronavirus be for you (how seriously ill do you think you will be)?**Not severe [*] [*] [*] [*] [*] [*] [*] Very severe  Please click CONTINUE to proceed |
| Page 8  Variable:  Preparedness and Perceived self-efficacy | **Next, we would like to know about you own practices related to the novel coronavirus.**  **I know how to protect myself from coronavirus**  Not at all [*] [*] [*] [*] [*] [*] [*] Very much so  **For me avoiding an infection with the novel coronavirus in the current situation is…**Extremely difficult [*] [*] [*] [*] [*] [*] [*] Extremely easy  Please click CONTINUE to proceed |
| Page 9    Variable: Prevention – own behaviours      [Random order of items; except “Another preventive measure”] | **Which of the following measures have you taken to prevent infection from the novel coronavirus?**  Please indicate for all measures below whether you have already taken them.  **Hand washing for at least 20 seconds** [*] Yes  [*] No  [*] Does not apply  **Avoiding touching your eyes, nose, and mouth with unwashed hands** [Answer scheme, see above]  **Use of disinfectants to clean hands when soap and water was not available for**  **washing hands** [Answer scheme, see above]  **Staying home when you were sick or when you had a cold** [Answer scheme, see above]  **Herbal supplements** [Answer scheme, see above]  **Covering your mouth and nose when you cough or sneeze** [Answer scheme, see above]  **Using caution when opening letters** [Answer scheme, see above]  **Getting the flu vaccine** [Answer scheme, see above]  **Wearing a face mask** [Answer scheme, see above]  **Using antibiotics** [Answer scheme, see above]  **Using homeopathic remedies** [Answer scheme, see above]  **Physical distancing (keeping minimum 2 metres between you and other persons outside your household)** [Answer scheme, see above]  **Self-isolation** [Answer scheme, see above]  **Disinfecting surfaces**[Answer scheme: see “Hand washing”]  **Disinfecting the mobile phone**[Answer scheme: see “Hand washing”]  **Eating garlic, ginger, lemon** [Answer scheme: see “Hand washing”]  **Another preventive measure, please specify**…  **Not seeing my family living outside my own home is**Impossible [*] [*] [*] [*] [*] [*] [*] Very easy to do  **Not seeing my friends is**Impossible [*] [*] [*] [*] [*] [*] [*] Very easy to do  Please click CONTINUE to proceed |
| Page 10  Variable: Knowledge and self-assessed adherence to preventive measures      [Random order of items; except “Another preventive measure”] | **I follow the recommendations from authorities in my country to prevent spread of novel coronavirus.**Not at all [*] [*] [*] [*] [*] [*] [*] Very much so  **Which of the following are effective measures to prevent the spread and infection of the novel coronavirus?**  Please evaluate all preventive measures listed below.  **Hand washing for at least 20 seconds** [*] Yes  [*] No  [*] Don’t know  **Avoiding touching your eyes, nose, and mouth with unwashed hands**[Answer scheme: see “Hand washing”]  **Use of disinfectants to clean hands when soap and water is not available for washing hands**[Answer scheme: see “Hand washing”]  **Staying home when you are sick or when you have a cold**[Answer scheme: see “Hand washing”]  **Herbal supplements**[Answer scheme: see “Hand washing”]  **Covering your mouth when you cough**[Answer scheme: see “Hand washing”]  **Using caution when opening letters**[Answer scheme: see “Hand washing”]  **Getting the flu vaccine**[Answer scheme: see “Hand washing”]  **Wearing a face mask**[Answer scheme: see “Hand washing”]  **Using antibiotics**[Answer scheme: see “Hand washing”]  **Using homeopathic remedies**[Answer scheme: see “Hand washing”]  **Physical distancing (keeping minimum 2 metres between you and other persons outside your household)**[Answer scheme: see “Hand washing”]  **Self-isolation**[Answer scheme: see “Hand washing”]  **Disinfecting surfaces**[Answer scheme: see “Hand washing”]  **Disinfecting the mobile phone**[Answer scheme: see “Hand washing”]  **Eating garlic, ginger, lemon** [Answer scheme: see “Hand washing”]  **Another preventive measure, please specify…**  [*] Yes  [*] No  [*] Don’t know  Please click CONTINUE to proceed |
| Page 11  Variable:  Affect        [Random order of items] | **Please choose one option per row below. The novel coronavirus to me feels ...**  close to me [*] [*] [*] [*] [*] [*] [*] far away from me  New [*] [*] [*] [*] [*] [*] [*] Old  Spreading slowly [*] [*] [*] [*] [*] [*] [*] Spreading fast  Something I think about all the time [*] [*] [*] [*] [*] [*] [*] Something I almost never think about  Fear-inducing [*] [*] [*] [*] [*] [*] [*] Not fear-inducing  Media hyped [*] [*] [*] [*] [*] [*] [*] Not media hyped  Worrying [*] [*] [*] [*] [*] [*] [*] Not worrying  Something that makes me feel helpless [*] [*] [*] [*] [*] [*] [*] Something I am able to combat with my own action  Stressful [*] [*] [*] [*] [*] [*] [*] Not stressful  Something that is making me depressed [*] [*] [*] [*] [*] [*] [*] Something that does not affect my mood  Please click CONTINUE to proceed |
| Page 12  Variable: Trust in sources of information    [Random order of items, except other]  [employer and health insurance are no compulsory items] | **How much do you trust the following sources of information in their reporting about the novel coronavirus?**  **Public television stations**Very little trust [*] [*] [*] [*] [*] [*] [*] A great deal of trust  **Daily or weekly newspapers** [Answer scheme, see above]  **Conversations with family and friends** [Answer scheme, see above]  **Conversations with colleagues** [Answer scheme, see above]  **Consultation with health workers** [Answer scheme, see above]  **Private television stations** [Answer scheme, see above]  **Websites or online news pages** (e.g.) [Answer scheme, see above]  **Social media (e.g. Facebook, Twitter, YouTube, WhatsApp)** [Answer scheme, see above]  **Private radio stations** [Answer scheme, see above]  **Public radio stations** [Answer scheme, see above]  **Official, government press releases**[Answer scheme, see above]  **Medical institutions press releases**[Answer scheme, see above]  **Celebrities and social media influencers**[Answer scheme, see above]  **Other sources, namely: _____**  Please click CONTINUE to proceed |
| Page 13  Variable: Use of sources of information    [Random order of items; except other]  [employer, health insurance, and other are no compulsory items] | **How often do you use the following sources of information to stay informed about the novel coronavirus?**  **Public television stations** Never [*] [*] [*] [*] [*] [*] [*] Very often  **Daily or weekly newspapers** [Answer scheme, see above]  **Conversations with family and friends** [Answer scheme, see above]  **Conversations with colleagues** [Answer scheme, see above]  **Private television stations** [Answer scheme, see above]  **Websites or online news pages (e.g. web.de, t-online.de)** [Answer scheme, see above]  **Social media (e.g. Facebook, Twitter, YouTube, WhatsApp)** [Answer scheme, see above]  **Private radio stations** [Answer scheme, see above]  **Public radio stations** [Answer scheme, see above]  **Official, government press releases**[Answer scheme, see above]  **Medical institutions press releases**[Answer scheme, see above]  **Celebrities and social media influencers**[Answer scheme, see above]  **Other sources, namely: _____**  **The type of information I need the most, relates to…**  **Symptoms of novel coronavirus** [*] Yes [*] No  **How I can protect myself and my family against the novel coronavirus** [*] Yes [*] No  **Personal stories from other people on how they cope with the pandemic situation** [*] Yes [*] No  **Scientific progress in development of a vaccine or treatment against novel coronavirus** [*] Yes [*] No  **How I can take care of a person who belongs to a risk group** [*] Yes [*] No  **How I can best take care of my children’s school education** [*] Yes [*] No  **How the novel coronavirus is different from other diseases such as flu**[*] Yes [*] No  **The pandemic evolution in the world**[*] Yes [*] No  **The pandemic evolution in Federation FBiH**[*] Yes [*] No  **Information about authorities’ decisions**[*] Yes [*] No  **How I will be impacted economically by the pandemic**[*] Yes [*] No  **How to maintain my mental health during the isolation**[*] Yes [*] No  **How to maintain my social contact despite the physical distancing** [*] Yes [*] No  **Other, please specify…**  Please click CONTINUE to proceed |
| Page 14  Variable: Frequency of Information | **How often do you inform yourself about the novel coronavirus?**  Never [*] [*] [*] [*] [*] [*] [*] Several times a day  Please click CONTINUE to proceed |
| Page 15  Variable: Trust in institutions (perceptions)    [Random order of items] | **How much confidence do you have in the below individuals and organizations that they can handle the novel coronavirus well?**  **Your own family doctor**Very low confidence [*] [*] [*] [*] [*] [*] [*] Very high confidence / Not applicable [*]  **Your company physician (if applicable)** [Answer scheme, see above]  **Your Employer (if applicable)** [Answer scheme, see above]  **Media** [Answer scheme, see above]  **Other opinion leaders**[Answer scheme, see above]  **Hospitals** [Answer scheme, see above]  **Ministry of Health** [Answer scheme, see above]  **Local Public Health Authority** [Answer scheme, see above]  **Medical professional associations (e.g. )** [Answer scheme, see above]  **Schools** [Answer scheme, see above]  **Universities** [Answer scheme, see above]  **Kindergartens** [Answer scheme, see above]  **Public transportation companies** [Answer scheme, see above]  **Government**[Answer scheme, see above]  **Police**[Answer scheme, see above]  **Army**[Answer scheme, see above]  **Church**[Answer scheme, see above]  **The president**[Answer scheme, see above]  **Other, please specify…**  Please click CONTINUE to proceed |
| Page 16    Variable:  Policies, interventions (perceptions)    [Random order of items] | **Please now give your opinion on the following statements.**  **If a vaccine becomes available and is recommended for me, I would get it.**Strongly disagree [*] [*] [*] [*] [*] [*] [*] Strongly agree  **In the event of an outbreak it’s appropriate to avoid certain people on the basis of their country of origin.**[See answer scheme above.]  **The government should be allowed to force people into self-isolation if they are infected**[See answer scheme above.]  **The government should be allowed to force people into self-isolation if they have been in contact with a person who was infected**[See answer scheme above.]  **The government should restrict access to the Internet and social media to combat the spread of misinformation about the novel coronavirus.**[See answer scheme above.]  **From now on, anyone moving in public areas should be required to wear a face mask** [See answer scheme above.]  **More tests for coronavirus infection should be carried out in the population** [See answer scheme above.]  **I think that the restrictions currently being implemented are greatly exaggerated.**[See answer scheme above.]  **It should only be allowed to leave your house for professional, health or urgent reasons.**[See answer scheme above.]  Please click CONTINUE to proceed |
| Page 17    Variable:  Conspiracies (perceptions) | **Please consider the decisions that are made in your country to reduce spread of the novel coronavirus:**  **I think that….**  **…many very important things happen in the world, which the public is never informed about** certainly not true [*] [*] [*] [*] [*] [*] [*]certainly true  **… politicians usually do not tell us the true motives for their decisions**[See answer scheme above.]  **… government agencies closely monitor all citizens**[See answer scheme above.]  **… events which superficially seem to lack a connection are often the result of secret activities**[See answer scheme above.]  **… there are secret organizations that greatly influence political decisions**[See answer scheme above.]  Please click CONTINUE to proceed |
| Page 18    Variable:  resilience (perceptions) | **Please consider your experience during the novel coronavirus pandemic:**  **I have a hard time making it through stressful events**Strongly disagree [*] [*] [*] [*] [*] [*] [*] Strongly agree  **It does not take me long to recover from a stressful event**[See answer scheme above.]  **It is hard for me to snap back when something bad happens**[See answer scheme above.]  Please click CONTINUE to proceed |
| Page 19    Variable:  Fairness (perceptions) | **Please consider the decisions that are made in your country to reduce spread of the novel coronavirus:**  **I think the decisions are fair**Strongly disagree [*] [*] [*] [*] [*] [*] [*] Strongly agree  **I would convince others that the decisions are right**Strongly disagree [*] [*] [*] [*] [*] [*] [*] Strongly agree  Please click CONTINUE to proceed |
| Page 20  Lifting restrictions (pandemic transition phase) | **There is currently a discussion about how some restrictions can be changed after the first peak of the novel coronavirus. Please give your opinion on the following statements.**  **After the peak of the pandemic, only people over the age of 70 should stay at home**Strongly disagree [*] [*] [*] [*] [*] [*] [*] Strongly agree  **After the peak of the pandemic, the restrictions should be upheld in towns and cities, but not rural areas**[See answer scheme above.]  **After the peak of the pandemic, the smaller children should be able to return to school, but not the older ones who can take care of themselves at home**[See answer scheme above.]  **After the peak of the pandemic, restrictions should be lifted in the counties where less people are infected, but not in counties where more people are infected**[See answer scheme above.]  **Current restrictions should not be lifted, even beyond the peak of the pandemic**[See answer scheme above.]  **After the peak of the pandemic, everyone should be obligated to wear a mask in public**. [See answer scheme above.]  **After the peak of the pandemic, obligatory mass testing is an acceptable way to identify infected persons**[See answer scheme above.]  **After the peak of the pandemic,] I will continue to live up to restrictions, even if they are no longer formal recommendations from my government** [See answer scheme above.]  Please click CONTINUE to proceed |
| Page 21    Variable: Behaviour  [Integrate in randomized order of the policy items]      [Random order of items] | **Have** you **done** the following during the pandemic**…?**  **Bought food supplies on a large scale**  [*] I already did that  [*] I plan to do that  [*] I don’t plan to do that  **Bought other everyday things on a large scale**[See answer scheme above.]  **Avoided people who come from countries where coronavirus cases have occurred, such as China or Italy**  **Exercised less than I usually do**[See answer scheme above.]  **Drank more alcohol than I usually do**[See answer scheme above.]  **Ate more unhealthy food than I usually do**[See answer scheme above.]  **Avoided going to the doctor with issues that could be postponed, e.g. vaccination or a check-up** [See answer scheme above.]  **Asked family members or friends not to visit me**[See answer scheme above.]  **Decided that my child could not meet with a friend**[See answer scheme above.]  **Bought drugs that I heard that are good for treating COVID-19**[See answer scheme above.]  **Bought personal protection equipment (masks, gloves)**[See answer scheme above.]  Please click CONTINUE to proceed |
| Page 22    Variable: Worry    [Random order of items; except other] | **Crises often involve fears and worries. Please let us know:**  **At the moment, how much do you worry about:**  **losing someone I love** Don’t worry at all [*] [*] [*] [*] [*] [*] [*] worry a lot  **health system being overloaded**[See answer scheme above.]  **my own mental health**[See answer scheme above.]  **my own physical health**[See answer scheme above.]  **my loved ones’ health**[See answer scheme above.]  **restricted liberty of movement**[See answer scheme above.]  **loosing vacation opportunities** [See answer scheme above.]  **small companies running out of business**[See answer scheme above.]  **economic recession in my country**[See answer scheme above.]  **restricted access to food supplies**[See answer scheme above.]  **becoming unemployed**[See answer scheme above.]  **not being able to pay my bills**[See answer scheme above.]  **not be able to visit people who depend on me** [See answer scheme above.]  **having to defend a decision not to participate in a social event which my family or friends expect me to attend** [See answer scheme above.]  **other:** ____  Please click CONTINUE to proceed |
| Page 23    Variable: Rumors | **Have you encountered information on the novel coronavirus where you found it hard to decide whether it was right or wrong? For example, information about ways to prevent the disease or to recover.**  **Please write this information in the boxes below. You can provide up to three types of information. Please use a separate box for each type of information.**  **Please note that on the next page you will receive links to trustworthy information about the novel coronavirus.**  **If you cannot or do not want to answer this question, scroll down and click No.**  **Information: _________**  **Information: _________**  **Information: _________**  **[*] No, I have not heard or read any information that I am unsure of.**  Please click CONTINUE to proceed |
| Page 24  Debriefing | Debriefing  Thank you very much!  Your participation provides valuable insights for all of us to react appropriately in the current novel coronavirus situation and to reach all citizens with useful information in a timely manner.  For information about the novel coronavirus**,** please visit the following websites: https://www.zzjzFBiH.ba/covid-19/  If you have any questions, please contact us by mail: covid-19@zzjzFBiH.ba  If you have changed your opinion and would like to withdraw your consent to use your data, please click on “Withdraw my consent”.  [*] Withdraw my consent  Please click CONTINUE to finish the survey |
